# 2-(4-Acetamido­benzene­sulfonamido)-3-methyl­butanoic acid

**DOI:** 10.1107/S1600536810016119

**Published:** 2010-05-08

**Authors:** Shahzad Sharif, Haffsah Iqbal, Islam Ullah Khan, Peter John, Edward R. T. Tiekink

**Affiliations:** a Materials Chemistry Laboratory, Department of Chemistry, Government College, University, Lahore 54000, Pakistan; b Department of Chemistry, University of Malaya, 50603 Kuala Lumpur, Malaysia

## Abstract

In the title compound, C_13_H_18_N_2_O_5_S, the benzene ring and the acetamide group are almost coplanar [dihedral angle = 5.6 (3)°], and the amine group projects almost vertically from this plane [C—C—S—N = −84.5 (7)°]. A short intra­molecular C—H⋯O contact occurs. In the crystal, O—H⋯O, N—H⋯O and N—H⋯(O,O) hydrogen bonds lead to a three-dimensional network. One of the methyl groups of the isopropyl residue is disordered over two orientations in a 0.747 (16):0.253 (16) ratio.

## Related literature

For background to the pharmacological uses of sulfonamides, see: Korolkovas (1988 ▶); Mandell & Sande (1992 ▶). For related structures, see: Sharif *et al.* (2010 ▶); Khan *et al.* (2010 ▶).
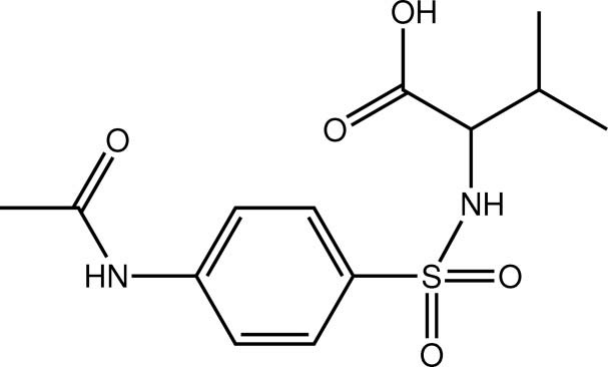


## Experimental

### 

#### Crystal data

C_13_H_18_N_2_O_5_S
*M* *_r_* = 314.35
Orthorhombic, 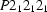

*a* = 5.1649 (13) Å
*b* = 14.724 (5) Å
*c* = 20.688 (7) Å
*V* = 1573.2 (8) Å^3^
*Z* = 4
Mo *K*α radiation
μ = 0.23 mm^−1^
*T* = 293 K
0.39 × 0.09 × 0.07 mm

#### Data collection

Bruker APEXII CCD diffractometer
7356 measured reflections
1647 independent reflections
1083 reflections with *I* > 2σ(*I*)
*R*_int_ = 0.075

#### Refinement

*R*[*F* ^2^ > 2σ(*F* ^2^)] = 0.053
*wR*(*F* ^2^) = 0.209
*S* = 1.16
1647 reflections
205 parameters
2 restraints
H atoms treated by a mixture of independent and constrained refinement
Δρ_max_ = 0.49 e Å^−3^
Δρ_min_ = −0.52 e Å^−3^

### 

Data collection: *APEX2* (Bruker, 2007 ▶); cell refinement: *SAINT* (Bruker, 2007 ▶); data reduction: *SAINT*; program(s) used to solve structure: *SHELXS97* (Sheldrick, 2008 ▶); program(s) used to refine structure: *SHELXL97* (Sheldrick, 2008 ▶); molecular graphics: *ORTEP-3* (Farrugia, 1997 ▶) and *DIAMOND* (Brandenburg, 2006 ▶); software used to prepare material for publication: *publCIF* (Westrip, 2010 ▶).

## Supplementary Material

Crystal structure: contains datablocks global, I. DOI: 10.1107/S1600536810016119/hb5434sup1.cif

Structure factors: contains datablocks I. DOI: 10.1107/S1600536810016119/hb5434Isup2.hkl

Additional supplementary materials:  crystallographic information; 3D view; checkCIF report

## Figures and Tables

**Table 1:** Hydrogen-bond geometry (Å, °)

*D*—H⋯*A*	*D*—H	H⋯*A*	*D*⋯*A*	*D*—H⋯*A*
C3—H3⋯O3	0.93	2.25	2.848 (11)	122
O5—H5o⋯O3^i^	0.93	1.66	2.591 (9)	176
N1—H1n⋯O1^ii^	0.86 (5)	2.34 (7)	3.147 (9)	157 (6)
N2—H2n⋯O2^iii^	0.86 (3)	2.37 (3)	3.184 (8)	158 (6)
N2—H2n⋯O4	0.86 (3)	2.35 (6)	2.767 (9)	110 (5)

Symmetry codes: (i) 
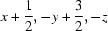
; (ii) 
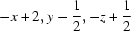
; (iii) 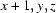.

**Fig. 1.**
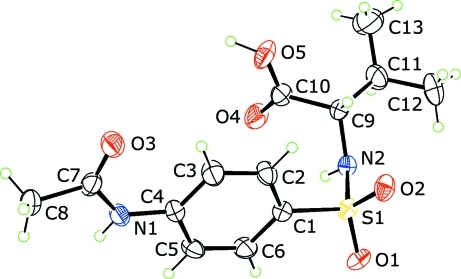
The molecular structure of (I) showing displacement ellipsoids at the 35% probability level.

**Fig. 2.**
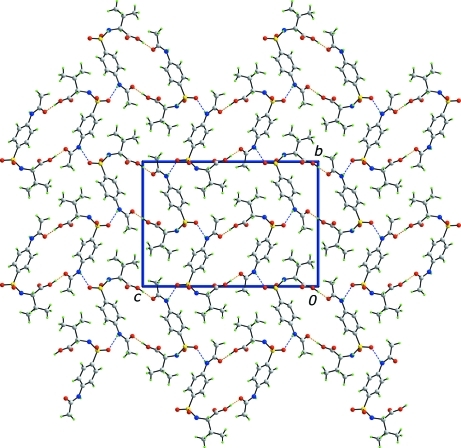
A view in projection down the a axis of the unit cell contents for (I). The O–H···O and N–H···O hydrogen bonds are shown as orange and blue dashed lines, respectively. Colour code: S, yellow; O, red; N, blue; C, grey; and H, green.

## supplementary crystallographic information

### Comment

Sulfonamide drugs are widely used for the treatment of certain infections caused by Gram-positive and Gram-negative microorganisms, some fungi, and certain protozoa (Korolkovas, 1988; Mandell & Sande, 1992). In continuation of structural investigations of sulfonamides (Sharif *et al.*, 2010; Khan *et al.*, 2010), herein, the crystal structure of title compound, (I), is described.

The structure analysis of (I), Fig. 1, shows that the acetamide group is co-planar with the benzene ring to which it is attached; the dihedral angle = 5.6 (3) °. This conformation is stabilised by an intramolecular C–H···O contact, Table 1. While the S atom also lies in this plane [the S1–C1–C2–C3 torsion angle = 176.8 (7) °], the sulfonamido-O atoms lie to one side [being displaced by 0.386 (5) Å for atom O1 and 0.555 (6) Å for O2] and the amine substituent to project almost vertically to the other [the N2–S1–C1–C2 torsion angle = -84.5 (7) °]. Within the amine group, the carboxylic acid group is folded back to lie over the benzene ring; the dihedral angle between the two planes = 34.2 (5) °.

The crystal structure is stabilised by O–H···O and N–H···O hydrogen bonding interactions, Table 1. Thus, the carboxylic acid-hydroxyl group forms a donor interaction to the amide-carbonyl, and each of the N—H atoms forms a donor interaction to a sulfonamido-O; an intramolecular interaction formed between the N2—H and carboxylic acid-carbonyl group is also noted, Table 1. The result of the hydrogen bonding just described is the formation of a 3-D network, Fig. 2.

### Experimental

To 2-amino-3-methylbutanoic acid (0.339 g, 2.8 mmol ) in distilled water (10 ml) was added 4-acetylaminobenzenesulfonyl chloride (0.7 g, 2.8 mmol) with stirring at room temperature, while maintaining the pH of the reaction mixture at 8 using 3% sodium carbonate. The progress of the reaction was monitored by TLC. On completion of reaction, the pH was adjusted to 3.0 by slow addition 3 N HCl.  The precipitate formed in this way was washed with water, dried and recrystalized from methanol and ethyl acetate mixture (50:50 v/v) to yield colourless prisms of (I); m. pt. 510 K.

### Refinement

The O- and C-bound H atoms were geometrically placed (O–H = 0.93 Å; C–H = 0.93–0.98 Å) and refined as riding with *U_iso_*(H) = 1.2-1.5*U_eq_*(O,*C*). The N-bound H atoms were refined with the distance restraint N–H = 0.86±0.01 Å, and with *U_iso_*(H) = 1.2*U_eq_*(N). High thermal motion was noted in the *iso*-propyl substituent and it proved possible to resolve two positions for one of the methyl groups. Anisotropic refinement (constrained to be equivalent for the components of the disorder by the EADP command in SHELXL-97) showed the major component of the disorder had a site occupancy factor = 0.747 (16). In the absence of significant anomalous scattering effects, 1130 Friedel pairs were averaged in the final refinement.

### Crystal data

**Table:**

C_13_H_18_N_2_O_5_S	*F*(000) = 664
*M**_r_* = 314.35	*D*_x_ = 1.327 Mg m^−^^3^
Orthorhombic, *P*2_1_2_1_2_1_	Mo *K*α radiation, λ = 0.71073 Å
Hall symbol: P 2ac 2ab	Cell parameters from 815 reflections
*a* = 5.1649 (13) Å	θ = 3.0–18.5°
*b* = 14.724 (5) Å	µ = 0.23 mm^−^^1^
*c* = 20.688 (7) Å	*T* = 293 K
*V* = 1573.2 (8) Å^3^	Prism, colourless
*Z* = 4	0.39 × 0.09 × 0.07 mm

### Data collection

**Table:**

Bruker APEXII CCD diffractometer	1083 reflections with *I* > 2σ(*I*)
Radiation source: fine-focus sealed tube	*R*_int_ = 0.075
graphite	θ_max_ = 25.0°, θ_min_ = 1.7°
φ and ω scans	*h* = −6→5
7356 measured reflections	*k* = −14→17
1647 independent reflections	*l* = −24→24

### Refinement

**Table:**

Refinement on *F*^2^	Primary atom site location: structure-invariant direct methods
Least-squares matrix: full	Secondary atom site location: difference Fourier map
*R*[*F*^2^ > 2σ(*F*^2^)] = 0.053	Hydrogen site location: inferred from neighbouring sites
*wR*(*F*^2^) = 0.209	H atoms treated by a mixture of independent and constrained refinement
*S* = 1.16	*w* = 1/[σ^2^(*F*_o_^2^) + (0.1138*P*)^2^] where *P* = (*F*_o_^2^ + 2*F*_c_^2^)/3
1647 reflections	(Δ/σ)_max_ < 0.001
205 parameters	Δρ_max_ = 0.49 e Å^−^^3^
2 restraints	Δρ_min_ = −0.52 e Å^−^^3^

### Special details

**Table:**

Geometry. All s.u.'s (except the s.u. in the dihedral angle between two l.s. planes) are estimated using the full covariance matrix. The cell s.u.'s are taken into account individually in the estimation of s.u.'s in distances, angles and torsion angles; correlations between s.u.'s in cell parameters are only used when they are defined by crystal symmetry. An approximate (isotropic) treatment of cell s.u.'s is used for estimating s.u.'s involving l.s. planes.
Refinement. Refinement of *F*^2^ against ALL reflections. The weighted *R*-factor wR and goodness of fit *S* are based on *F*^2^, conventional *R*-factors *R* are based on *F*, with *F* set to zero for negative *F*^2^. The threshold expression of *F*^2^ > 2σ(*F*^2^) is used only for calculating *R*-factors(gt) etc. and is not relevant to the choice of reflections for refinement. *R*-factors based on *F*^2^ are statistically about twice as large as those based on *F*, and *R*- factors based on ALL data will be even larger.

### Fractional atomic coordinates and isotropic or equivalent isotropic displacement parameters (Å^2^)

**Table:**

	*x*	*y*	*z*	*U*_iso_*/*U*_eq_	Occ. (<1)
S1	0.6472 (3)	0.98522 (14)	0.24127 (8)	0.0374 (6)	
O1	0.7761 (10)	0.9900 (4)	0.3028 (2)	0.0474 (15)	
O2	0.3793 (9)	1.0094 (4)	0.2371 (3)	0.0484 (14)	
O3	0.4852 (13)	0.6006 (4)	0.0597 (3)	0.0627 (19)	
O4	1.0931 (11)	0.9940 (5)	0.0904 (3)	0.068 (2)	
O5	0.7406 (11)	0.9868 (5)	0.0302 (3)	0.0618 (19)	
H5O	0.8216	0.9540	−0.0023	0.093*	
N1	0.8024 (12)	0.6160 (5)	0.1338 (3)	0.0423 (17)	
H1N	0.947 (8)	0.593 (5)	0.146 (4)	0.051*	
N2	0.8017 (12)	1.0547 (4)	0.1940 (3)	0.0347 (15)	
H2N	0.963 (4)	1.041 (5)	0.194 (3)	0.042*	
C1	0.6850 (14)	0.8749 (5)	0.2107 (4)	0.0341 (17)	
C2	0.5207 (16)	0.8443 (6)	0.1626 (4)	0.044 (2)	
H2	0.3858	0.8810	0.1483	0.053*	
C3	0.5570 (17)	0.7596 (6)	0.1360 (4)	0.051 (2)	
H3	0.4462	0.7395	0.1036	0.061*	
C4	0.7560 (14)	0.7038 (5)	0.1568 (4)	0.0381 (19)	
C5	0.9198 (15)	0.7362 (6)	0.2053 (4)	0.045 (2)	
H5	1.0537	0.6994	0.2201	0.054*	
C6	0.8875 (16)	0.8207 (6)	0.2314 (4)	0.047 (2)	
H6	1.0011	0.8417	0.2629	0.056*	
C7	0.6686 (18)	0.5689 (6)	0.0892 (4)	0.045 (2)	
C8	0.7567 (19)	0.4720 (5)	0.0793 (4)	0.057 (2)	
H8A	0.6125	0.4316	0.0850	0.086*	
H8B	0.8891	0.4575	0.1102	0.086*	
H8C	0.8245	0.4652	0.0364	0.086*	
C9	0.7105 (16)	1.0683 (6)	0.1282 (3)	0.0403 (19)	
H9	0.5295	1.0486	0.1256	0.048*	
C10	0.8709 (15)	1.0112 (6)	0.0812 (3)	0.0429 (19)	
C11	0.724 (2)	1.1699 (7)	0.1107 (5)	0.071 (3)	0.747 (16)
H11	0.9013	1.1893	0.1209	0.086*	0.747 (16)
C12	0.550 (3)	1.2240 (7)	0.1542 (6)	0.103 (5)	0.747 (16)
H12A	0.5831	1.2876	0.1483	0.154*	0.747 (16)
H12B	0.5821	1.2077	0.1984	0.154*	0.747 (16)
H12C	0.3724	1.2113	0.1437	0.154*	0.747 (16)
C13	0.683 (4)	1.1929 (11)	0.0441 (7)	0.109 (7)	0.747 (16)
H13A	0.5309	1.1626	0.0286	0.164*	0.747 (16)
H13B	0.8302	1.1742	0.0190	0.164*	0.747 (16)
H13C	0.6608	1.2574	0.0402	0.164*	0.747 (16)
C11A	0.724 (2)	1.1699 (7)	0.1107 (5)	0.071 (3)	0.253 (16)
H11A	0.6178	1.1695	0.0715	0.086*	0.253 (16)
C12A	0.550 (3)	1.2240 (7)	0.1542 (6)	0.103 (5)	0.253 (16)
H12D	0.6523	1.2566	0.1851	0.154*	0.253 (16)
H12E	0.4339	1.1837	0.1764	0.154*	0.253 (16)
H12F	0.4513	1.2663	0.1288	0.154*	0.253 (16)
C13A	0.928 (14)	1.213 (3)	0.088 (2)	0.109 (7)	0.253 (16)
H13D	0.9508	1.1990	0.0433	0.164*	0.253 (16)
H13E	1.0795	1.1945	0.1118	0.164*	0.253 (16)
H13F	0.9041	1.2774	0.0933	0.164*	0.253 (16)

### Atomic displacement parameters (Å^2^)

**Table:**

	*U*^11^	*U*^22^	*U*^33^	*U*^12^	*U*^13^	*U*^23^
S1	0.0357 (10)	0.0429 (11)	0.0336 (9)	−0.0026 (9)	0.0032 (8)	−0.0080 (8)
O1	0.055 (3)	0.058 (4)	0.029 (3)	−0.006 (3)	−0.001 (2)	−0.008 (3)
O2	0.032 (3)	0.058 (4)	0.055 (3)	0.003 (3)	0.006 (2)	−0.013 (3)
O3	0.078 (4)	0.049 (4)	0.061 (4)	0.014 (4)	−0.028 (4)	−0.016 (3)
O4	0.047 (4)	0.104 (6)	0.054 (4)	0.028 (4)	−0.001 (3)	−0.015 (4)
O5	0.052 (4)	0.080 (5)	0.053 (3)	0.006 (3)	−0.001 (3)	−0.034 (4)
N1	0.045 (4)	0.034 (4)	0.048 (4)	0.008 (3)	−0.007 (3)	−0.001 (3)
N2	0.032 (4)	0.033 (3)	0.039 (3)	−0.002 (3)	0.004 (3)	−0.002 (3)
C1	0.031 (4)	0.035 (4)	0.036 (4)	−0.003 (3)	0.000 (3)	−0.002 (3)
C2	0.036 (4)	0.040 (5)	0.056 (5)	0.005 (4)	−0.011 (4)	−0.006 (4)
C3	0.048 (5)	0.045 (5)	0.059 (6)	0.003 (4)	−0.020 (4)	−0.014 (5)
C4	0.037 (4)	0.034 (4)	0.043 (4)	0.004 (4)	0.001 (4)	0.000 (4)
C5	0.044 (5)	0.043 (5)	0.047 (5)	0.006 (4)	−0.013 (4)	0.008 (4)
C6	0.048 (5)	0.047 (5)	0.044 (5)	−0.003 (4)	−0.018 (4)	−0.004 (4)
C7	0.060 (6)	0.037 (5)	0.038 (4)	−0.004 (4)	0.006 (5)	−0.006 (4)
C8	0.077 (6)	0.039 (5)	0.054 (5)	0.010 (5)	−0.008 (5)	−0.011 (5)
C9	0.042 (5)	0.044 (5)	0.035 (4)	0.008 (4)	0.006 (3)	−0.001 (4)
C10	0.035 (5)	0.049 (5)	0.045 (4)	0.000 (4)	0.012 (4)	0.004 (4)
C11	0.108 (9)	0.041 (6)	0.064 (6)	0.021 (6)	0.020 (6)	0.014 (5)
C12	0.154 (13)	0.053 (7)	0.102 (9)	0.052 (8)	0.004 (9)	−0.011 (7)
C13	0.21 (2)	0.057 (9)	0.064 (10)	0.008 (12)	−0.005 (11)	0.004 (8)
C11A	0.108 (9)	0.041 (6)	0.064 (6)	0.021 (6)	0.020 (6)	0.014 (5)
C12A	0.154 (13)	0.053 (7)	0.102 (9)	0.052 (8)	0.004 (9)	−0.011 (7)
C13A	0.21 (2)	0.057 (9)	0.064 (10)	0.008 (12)	−0.005 (11)	0.004 (8)

### Geometric parameters (Å, °)

**Table:**

S1—O2	1.431 (5)	C8—H8B	0.9600
S1—O1	1.438 (5)	C8—H8C	0.9600
S1—N2	1.625 (6)	C9—C10	1.530 (10)
S1—C1	1.754 (8)	C9—C11A	1.541 (12)
O3—C7	1.220 (10)	C9—C11	1.541 (12)
O4—C10	1.191 (9)	C9—H9	0.9800
O5—C10	1.301 (9)	C11—C13	1.434 (17)
O5—H5O	0.9275	C11—C12	1.503 (14)
N1—C7	1.345 (10)	C11—H11	0.9800
N1—C4	1.398 (10)	C12—H12A	0.9600
N1—H1N	0.86 (5)	C12—H12B	0.9600
N2—C9	1.454 (9)	C12—H12C	0.9600
N2—H2N	0.86 (3)	C13—H13A	0.9600
C1—C6	1.383 (10)	C13—H13B	0.9600
C1—C2	1.383 (10)	C13—H13C	0.9600
C2—C3	1.377 (11)	C11A—C13A	1.32 (7)
C2—H2	0.9300	C11A—C12A	1.503 (14)
C3—C4	1.385 (11)	C11A—H11A	0.9800
C3—H3	0.9300	C12A—H12D	0.9600
C4—C5	1.395 (10)	C12A—H12E	0.9600
C5—C6	1.368 (11)	C12A—H12F	0.9600
C5—H5	0.9300	C13A—H13D	0.9600
C6—H6	0.9300	C13A—H13E	0.9600
C7—C8	1.512 (12)	C13A—H13F	0.9600
C8—H8A	0.9600		
			
O2—S1—O1	119.3 (3)	H8A—C8—H8C	109.5
O2—S1—N2	106.4 (4)	H8B—C8—H8C	109.5
O1—S1—N2	105.9 (3)	N2—C9—C10	110.1 (6)
O2—S1—C1	108.4 (3)	N2—C9—C11A	109.8 (7)
O1—S1—C1	108.2 (4)	C10—C9—C11A	111.0 (7)
N2—S1—C1	108.1 (3)	N2—C9—C11	109.8 (7)
C10—O5—H5O	119.8	C10—C9—C11	111.0 (7)
C7—N1—C4	128.6 (7)	N2—C9—H9	108.6
C7—N1—H1N	116 (6)	C10—C9—H9	108.6
C4—N1—H1N	114 (6)	C11A—C9—H9	108.6
C9—N2—S1	119.4 (5)	C11—C9—H9	108.6
C9—N2—H2N	110 (5)	O4—C10—O5	124.8 (7)
S1—N2—H2N	109 (5)	O4—C10—C9	122.4 (8)
C6—C1—C2	119.9 (7)	O5—C10—C9	112.8 (7)
C6—C1—S1	120.4 (6)	C13—C11—C12	111.1 (12)
C2—C1—S1	119.5 (6)	C13—C11—C9	116.7 (10)
C1—C2—C3	119.9 (8)	C12—C11—C9	110.2 (9)
C1—C2—H2	120.0	C13—C11—H11	106.0
C3—C2—H2	120.0	C12—C11—H11	106.0
C4—C3—C2	120.9 (8)	C9—C11—H11	106.0
C4—C3—H3	119.5	C13A—C11A—C12A	116 (2)
C2—C3—H3	119.5	C13A—C11A—C9	126 (3)
C3—C4—C5	118.1 (7)	C12A—C11A—C9	110.2 (9)
C3—C4—N1	124.7 (7)	C13A—C11A—H11A	99.3
C5—C4—N1	117.2 (7)	C12A—C11A—H11A	99.3
C6—C5—C4	121.4 (7)	C9—C11A—H11A	99.3
C6—C5—H5	119.3	C11A—C12A—H12D	109.5
C4—C5—H5	119.3	C11A—C12A—H12E	109.5
C5—C6—C1	119.7 (7)	H12D—C12A—H12E	109.5
C5—C6—H6	120.2	C11A—C12A—H12F	109.5
C1—C6—H6	120.2	H12D—C12A—H12F	109.5
O3—C7—N1	122.9 (8)	H12E—C12A—H12F	109.5
O3—C7—C8	121.8 (8)	C11A—C13A—H13D	109.5
N1—C7—C8	115.2 (8)	C11A—C13A—H13E	109.5
C7—C8—H8A	109.5	H13D—C13A—H13E	109.5
C7—C8—H8B	109.5	C11A—C13A—H13F	109.5
H8A—C8—H8B	109.5	H13D—C13A—H13F	109.5
C7—C8—H8C	109.5	H13E—C13A—H13F	109.5
			
O2—S1—N2—C9	−49.5 (6)	C4—N1—C7—C8	−174.9 (8)
O1—S1—N2—C9	−177.4 (6)	S1—N2—C9—C10	−99.4 (7)
C1—S1—N2—C9	66.8 (6)	S1—N2—C9—C11A	138.0 (7)
O2—S1—C1—C6	−153.6 (6)	S1—N2—C9—C11	138.0 (7)
O1—S1—C1—C6	−22.9 (7)	N2—C9—C10—O4	−31.3 (11)
N2—S1—C1—C6	91.4 (7)	C11A—C9—C10—O4	90.5 (11)
O2—S1—C1—C2	30.5 (7)	C11—C9—C10—O4	90.5 (11)
O1—S1—C1—C2	161.2 (6)	N2—C9—C10—O5	150.7 (7)
N2—S1—C1—C2	−84.5 (7)	C11A—C9—C10—O5	−87.5 (9)
C6—C1—C2—C3	0.8 (12)	C11—C9—C10—O5	−87.5 (9)
S1—C1—C2—C3	176.7 (7)	N2—C9—C11—C13	169.7 (13)
C1—C2—C3—C4	0.2 (13)	C10—C9—C11—C13	47.7 (16)
C2—C3—C4—C5	−0.4 (13)	C11A—C9—C11—C13	0(100)
C2—C3—C4—N1	178.2 (8)	N2—C9—C11—C12	−62.3 (12)
C7—N1—C4—C3	−0.9 (13)	C10—C9—C11—C12	175.7 (9)
C7—N1—C4—C5	177.7 (8)	C11A—C9—C11—C12	0(100)
C3—C4—C5—C6	−0.5 (12)	N2—C9—C11A—C13A	85 (3)
N1—C4—C5—C6	−179.2 (7)	C10—C9—C11A—C13A	−37 (3)
C4—C5—C6—C1	1.5 (12)	C11—C9—C11A—C13A	0(100)
C2—C1—C6—C5	−1.7 (12)	N2—C9—C11A—C12A	−62.3 (12)
S1—C1—C6—C5	−177.6 (6)	C10—C9—C11A—C12A	175.7 (9)
C4—N1—C7—O3	3.5 (13)	C11—C9—C11A—C12A	0(100)

### Hydrogen-bond geometry (Å, °)

**Table:**

*D*—H···*A*	*D*—H	H···*A*	*D*···*A*	*D*—H···*A*
C3—H3···O3	0.93	2.25	2.848 (11)	122
O5—H5o···O3^i^	0.93	1.66	2.591 (9)	176
N1—H1n···O1^ii^	0.86 (5)	2.34 (7)	3.147 (9)	157 (6)
N2—H2n···O2^iii^	0.86 (3)	2.37 (3)	3.184 (8)	158 (6)
N2—H2n···O4	0.86 (3)	2.35 (6)	2.767 (9)	110 (5)

Symmetry codes:  (i) *x*+1/2, −*y*+3/2, −*z*; (ii) −*x*+2, *y*−1/2, −*z*+1/2; (iii) *x*+1, *y*, *z*.
